# Biopsychosocial factors associated with physical activity among Resettlers of the former Soviet Union in Germany: a cross-sectional analysis

**DOI:** 10.1136/bmjopen-2024-086042

**Published:** 2024-12-09

**Authors:** Mary Achieng Ouma, Kenneth Juma, Christa Meisinger, Susanne Stolpe, Heiko Becher, Volker Franz Winkler, Andreas Deckert

**Affiliations:** 1 Bielefeld University School of Public Health, Bielefeld, Nordrhein-Westfalen, Germany; 2 Heidelberg Institute of Global Health, Heidelberg University, Heidelberg, Baden-Württemberg, Germany; 3 Sexual, Reproductive, Maternal, Newborn, Child and Adolescent Health Unit, African Population and Health Research Center, Nairobi, Kenya; 4 Epidemiology, Medical Faculty, University of Augsburg, Augsburg, Bayern, Germany; 5 Center for Clinical Epidemiology, Universitatsklinikum Essen, Essen, Germany

**Keywords:** SPORTS MEDICINE, Behavior, Cross-Sectional Studies

## Abstract

**Abstract:**

**Objective:**

Previous research suggests an overall lower cardiovascular disease mortality among ethnic German Resettlers from the Former Soviet Union. However, evidence points to a high burden of metabolic risk factors and chronic conditions among Resettlers, factors which are correlated to lower levels of physical activity. Thus, this study aims to assess factors associated with physical activity among Resettlers, by investigating the interplay between biopsychosocial factors and physical activity between men and women.

**Design:**

We conducted a cross-sectional study by administering questionnaires between 2011 and 2012. Data from 595 individuals were analysed, and total and extracurricular physical activity scores were generated. A backward stepwise linear regression was run to investigate the effect of various predictors on various physical activity domains.

**Setting:**

Augsburg, Germany.

**Participants:**

We targeted Resettlers who had moved to Augsburg, Germany between 1990 and 1999.

**Results:**

Disease and psychological distress were linked to decreasing physical activity in men. Higher socioeconomic status was correlated with increasing physical activity except for work-related physical activity for both men and women. Single women were less likely to report extracurricular activity, and so did women who reported a shorter duration of stay and men who smoked.

**Conclusions:**

Migration experience and culture account for gender-related sex differences in physical activity alongwith other interlocking social factors such as psychological stressors and health status among Resettlers. Individual psychological stressors may hinder or motivate physical activity, but physical activity can reverse the influence of such stressors on Resettlers. Understanding migrant health and experiences is crucial due to the migration influx and health disparities. However, data on this topic are scarce in Germany.

**STRENGTHS AND LIMITATIONS OF THIS STUDY**

- This is the most extensive survey completed within this German subpopulation, which is often hard to convince to take part in surveys.
- The questionnaire was bilingual and tailored to the subpopulations under study.
- Physical activity measurement gave examples of activities entailed in each domain, allowing for quick and simple understanding.
- Physical activity assessment was self-reported consequently compromising the reliability of the tool.
- The participants mostly answered all sections of the questionnaire; however, there were a few sections of the survey with a considerably higher proportion of missing data (eg, frequent pain: 12.3% missing).

## Background

 In Germany, more than 21 million documented people (26%) have a migrant background, implying that either they or at least one parent was born without German citizenship.^1^ The second-largest group of people with a migrant background in Germany are the so-called Resettlers. More than three million ethnic Germans started immigrating from Eastern and Central Europe in the 1950s. Most came after 1990 from countries of the Former Soviet Union (FSU).^2 3^

Regarding their health status, Resettlers can be considered a high-risk population because a high prevalence of risk factors such as insufficient physical activity, obesity, hyperlipidaemia and chronic diseases such as diabetes characterises them.^4^,^6^ Surprisingly, though, studies found lower overall mortality than the autochthone German population.^6 7^ Physical inactivity is one of the leading causes of and a common modifiable risk factor for non-communicable diseases burdening the Resettler population and the healthcare system globally.^4^,^68^ In 2019, the WHO estimated that worldwide, about 17.9 million people (32% of global deaths) died from cardiovascular diseases (CVDs), and this proportion is expected to rise further.^9^ Between 2000 and 2019, deaths due to diabetes mellitus increased by 70%.^10^ Yet, about 1.4 billion people (28%) globally do not achieve the recommended minimum levels of physical activity.^9 11 12^ Highlighting sex disparities in physical activity, in 2016, it was reported from high-income countries that 23% of men and 32% of women did not meet the WHO physical activity standards.^13^

In 2010, Germany adopted the WHO’s global health recommendations on physical activity and implemented most health promotion strategies to combat the disease burden;^14^ however, only 46% of adults in Germany achieved the physical activity standard in 2018.^15^ While there is generally a dearth of information regarding the health status and behaviour of migrants, the proportion of migrants meeting physical activity recommendations is lower compared with the autochthonous population, and physical activity patterns are different from those of native Germans.^16 17^ For instance, in an urban German setting, only 20% of Turkish descendants remained physically active over several years.^18^ Nonetheless, there is a dearth of literature about how migratory contexts affect health.^19^

Alongside the biomedical differences and influence of physical inactivity, disparities in the health of migrants compared with the autochthonous populations have also been associated with the social determinants of health, that is, migrant minority status, reasons for migration, migration experience and the influence of the migration process on succeeding generations.^20^ Understanding migrant health and exploring their structural determinants is a pivotal step towards a better appreciation of immigrant health and the design of culturally sensitive interventions. Evidence from community-based research in the USA and Canada suggests the need to explore determinants of physical activity among migrants to eliminate physical activity barriers and promote health.^21^,^23^ There is value in understanding and contributing to the literature gap on the factors associated with the varying degrees of physical activity among Resettlers, who typically face complex social, economic and environmental conditions that can impede physical activity.^24^,^26^

The study aims to investigate the relationship between men’s and women’s physical activity and biopsychosocial factors, namely, sociodemographic, physiological, psychological, migration experience and health behaviour factors among Resettlers from the FSU in Germany. As in our analysis, generating more data and evidence expands the migration-health research agenda. It advances the understanding of factors that drive migrant health and how communities can effectively manage these issues. Such data could also be used to influence and support advocacy towards greater resource allocation and policies that address the health challenges and risks faced by resettled migrants in Germany and migrants across the globe.

## Methods

### Study design, sample, and data collection

We conducted a cross-sectional study targeting Resettlers from the FSU who immigrated to the Augsburg region in Germany between 1990 and 1999. The sample was captured from a register reconciliation of Resettlers who were still alive in 2010. A questionnaire was used for data collection in 2011 and 2012, investigating migration and family history, socioeconomic status, medical history, risk factors for ill health and physical activity. After excluding all individuals younger than 15 years of age, the questionnaire was administered to 3718 Resettlers. Out of these, 685 (18.4%) individuals completed the questionnaires. Finally, 595 (86.7%) questionnaires were available for analysis; in 89 (13.0%) cases, informed consent was missing, and one had no information on sex (see online supplemental figure 1 for participant’s flow chart). The study adhered to the Helsinki Declaration and was authorised by the medical association of the federal state Bavaria ethics committee. Comprehensive information on the participants, study design and procedures can be found elsewhere.^5^

### Measures

#### Dependent variables

In the questionnaire, three physical activity domains were assessed as follows:

1. ‘How often do you participate in sports? (For example, walking, running, swimming, football, tennis and gymnastics)’, denoting moderate to vigorous physical activity (MVPA), measured as ‘regularly more than 2 hours in the week’ (score 3), ‘regularly 1 to 2 hours in the week’ (score 2), ‘less than 1 hour in the week’ (score 1) and ‘no participation in sports’ (score 0).
2. ‘How often do you engage in physical activity? (For example, hiking, cycling, gardening and dancing)’, defining leisure-time physical activity (LPA), measured as ‘regularly more than 2 hours in the week’ (score 3), ‘regularly 1 to 2 hours in the week’ (score 2), ‘less than 1 hour in the week’ (score 1) and ‘not at all’ (score 0).
3. ‘How would you classify your main occupation?’ Representing work-related physical activity (WPA), measured as ‘vigorous physical work’ (score 3), ‘moderate physical work’ (score 2), ‘light physical work’ (score 1) and ‘no physical work’ (score 0).

The choice of using subjective measurements was because the questionnaire was designed to mainly evaluate the health status of the Resettlers, considering physical activity as one of the risk factors and to give a quick and simple understanding of physical activity domains/intensities to our sample.^27^

For the analysis, three physical activity outcome variables were utilised according to the scores of the three physical activity domains: WPA, extracurricular physical activity (EPA=MVPA+LPA) and total physical activity (TPA=MVPA+LPA+WPA). Histograms of the various domains are in the online supplemental tables 3-5.

#### Independent variables

Additionally, the independent variable ‘perceived control’^28^ was computed as the sum of the five-point Likert scales (never to always), assessing whether the participant perceived having the respective feeling within the last 4 weeks from these eight items: feeling happy, feeling calm, feeling energetic (coded 1–5); feeling worn-out, feeling sad, feeling anxious, feeling lonely and feeling doubtful (coded 5–1), so that the total score ranged from 8 to 40 (see online supplemental table 1).

For the variables ‘Body mass index’ and ‘Ethnicity of friends in Germany’, distinct categories with few observations were combined with other categories. Self-reported weight and height were utilised to determine the Body Mass Index, and the universally recognised WHO cut-off standard was used, as given in table 1.^29^

**Table 1:** Biodemographic characteristics of the study population

		Total		Men		Women	
**Demographics**		**n=595**	**100%**	**n=231**	**38.8%**	**n=364**	**61.2%**
Age	≤20 years	3	0.5	1	0.4	2	0.5
	21–30 years	11	1.8	5	2.2	6	1.6
	31–40 years	79	13.3	27	11.7	52	14.3
	41–50 years	120	20.2	44	19.0	76	20.9
	51–60 years	170	28.6	66	28.6	104	28.6
	61–70 years	112	18.8	46	19.9	66	18.1
	≥71 years	91	15.3	38	16.5	53	14.6
	Missing*	9	1.5	4	1.7	5	1.4
Marital status	Single	33	5.5	9	3.9	24	6.6
	Divorced/widowed	113	19.0	24	10.4	89	24.4
	Married	446	75.0	196	84.8	250	68.7
	Missing*	3	0.5	2	0.9	1	0.3
Educational level	University	127	21.3	46	19.9	81	22.3
	Upper secondary school	136	22.9	50	21.6	86	23.6
	Below upper secondary school	316	53.1	128	55.4	188	51.6
	Missing*	16	2.7	7	3.0	9	2.5
Employment status	Unemployed	45	7.6	13	5.6	32	8.8
	Retired	152	25.5	60	26.0	92	25.3
	Employed	396	66.6	158	68.4	238	65.4
	Missing*	2	0.3	0	0.0	2	0.5
**Physiologicalcharacteristics**							
Myocardial infarction	Yes	27	4.5	23	10.0	4	1.1
	No	559	93.9	207	89.6	352	96.7
	Missing*	9	1.5	1	0.4	8	2.2
Stroke	Yes	15	2.5	9	3.9	6	1.6
	No	574	96.5	221	95.7	353	97.0
	Missing*	6	1.0	1	0.4	5	1.4
Tumour	Yes	27	4.5	9	3.9	18	4.9
	No	563	94.6	220	95.2	343	94.2
	Missing*	5	0.8	2	0.9	3	0.8
Diabetes	Yes	74	12.4	27	11.7	47	12.9
	No	515	86.6	201	87.0	314	86.3
	Missing*	6	1.0	3	1.3	3	0.8
Body Mass Index	≥30 (obese)	225	37.8	108	46.8	117	32.1
	25–29.9 (overweight)	205	34.5	68	29.4	137	37.6
	<25 (under/normal weight)	122	20.5	33	14.3	89	24.5
	Missing*	43	7.2	22	9.5	21	5.8
Mobility restrictions	Mild to bedridden	346	58.2	127	55.0	219	60.2
	No	234	39.3	97	42.0	137	37.6
	Missing*	15	2.5	7	3.0	8	2.2
Frequent pain	Yes	213	35.8	73	31.6	140	38.5
	No	309	51.9	126	54.5	183	50.3
	Missing*	73	12.3	32	13.9	41	11.3

* Number of missing in relation to Nn=595 (not part of the categories).

### Patient and public involvement

The ‘Landsmannschaft der Deutschen aus Russland e.V.’ (the association of ethnic Germans from the FSU) was informed about the study and their feedback regarding the planning and conduct of the study and study materials was considered, which included the translation of the questionnaire. Participant recruitment was done in close consultation and collaboration with other representatives of the Resettlers and local physicians who were in contact with Resettlers and/or were Resettlers themselves. This enhanced response rates and eliminated language barriers. The association ‘Landsmannschaft der Deutschen aus Russland e.V.’ also supported the study by promoting it in their network (public events, newspapers, etc).

### Analysis

Data were analysed using IBM SPSS Statistics V.20 and Stata V.SE 15. Descriptive statistics illustrate the distribution of all factors by frequencies and percentages.

Multivariate linear regression models were fitted separately by sex to investigate the association between independent predictor variables (demographics, physiological characteristics, psychological stressors including the perceived control score, health behaviour and migration-related variables) and WPA, EPA and TPA scores. First, for each physical activity domain, linear regression with backward selection (stepwise removal of variables with p≥0.1, forcing in age and education as confounders) was conducted (see online supplemental table 2 for full models). Regarding missing variables, a complete case analysis was run. The choice of running a backward selection regression was based on the interest of developing models that explain the physical activity pattern best rather than being interested in the impact of a single variable (adjusted for confounders). We provide the results of the full linear regression models, which we used to investigate possible differences in the regression estimates resulting from the backward selection models.

## Results

### Sociodemographic characteristics

Overall, 364 women (61.2%) and 231 men (38.8%) participated in the study. The majority of respondents were above the age of 40 (82.9%) and were in their prime working years, reflected in 67% currently employed. The participants had a median age of 56 (IQR: 46–64).

In table 1, we see that diabetes was the most prevalent, with more women diagnosed than men, whereas more men reported a myocardial infarction compared with women. About 72.3% of the population were either overweight or obese, with more men being obese, while more women were overweight. A larger proportion of women had mobility restrictions.

Table 2 further reports the psychosocial and behavioural factors characterising the Resettlers. More men than women report not feeling at home, but more women than men report feeling worn-out. Nonetheless, a larger proportion of participants report not feeling anxious and lonely. Additionally, a considerable proportion of participants are mainly affected by financial difficulties and thoughts of previous stressful events, with more men (54.1%) reporting the former and women (51.4%) the latter.

**Table 2:** Psychosocial and behavioural characteristics of the study population

		Total		Men		Women	
		**n=595**	**100%**	**n=231**	**38.8%**	**n=364**	**61.2%**
**Psychological stressors**							
Feeling at home	No	67	11.3	38	16.5	29	8.0
	Mostly	219	36.8	73	31.6	146	40.1
	Yes	295	49.6	109	47.2	186	51.1
	Missing*	14	2.4	11	4.8	3	0.8
Feeling worn-out	No	227	38.2	110	47.6	117	32.1
	Sometimes	197	33.1	61	26.4	136	37.4
	Often	125	21.0	42	18.2	83	22.8
	Missing*	46	7.7	18	7.8	28	7.7
Feeling energetic	No	176	29.6	63	27.3	113	31.0
	Sometimes	154	25.9	56	24.2	98	26.9
	Often	197	33.1	89	38.5	108	29.7
	Missing*	68	11.4	23	10.0	45	12.4
Feeling calm	No	100	16.8	30	13.0	70	19.2
	Sometimes	131	22.0	46	19.9	85	23.4
	Often	321	53.9	139	60.2	182	50.0
	Missing*	43	7.2	16	6.9	27	7.4
Feeling anxious	No	416	69.9	181	78.4	235	64.6
	Sometimes	69	11.6	17	7.4	52	14.3
	Often	48	8.1	15	6.5	33	9.1
	Missing*	62	10.4	18	7.8	44	12.1
Feeling sad	No	275	46.2	125	54.1	150	41.2
	Sometimes	163	27.4	60	26.0	103	28.3
	Often	102	17.1	28	12.1	74	20.3
	Missing*	55	9.2	18	7.8	37	10.2
Feeling doubtful	No	347	58.3	153	66.2	194	53.3
	Sometimes	107	18.0	31	13.4	76	20.9
	Often	84	14.1	31	13.4	53	14.6
	Missing*	57	9.6	16	6.9	41	11.3
Feeling lonely	No	408	68.6	178	77.1	230	63.2
	Sometimes	74	12.4	16	6.9	58	15.9
	Often	59	9.9	20	8.7	39	10.7
	Missing*	54	9.1	17	7.4	37	10.2
Feeling happy	No	122	20.5	42	18.2	80	22.0
	Sometimes	185	31.1	79	34.2	106	29.1
	Often	225	37.8	88	38.1	137	37.6
	Missing*	63	10.6	22	9.5	41	11.3
Affected by family difficulties	No	366	61.5	158	68.4	208	57.1
	Yes	186	31.3	59	25.5	127	34.9
	Missing*	43	7.2	14	6.1	29	8.0
Affected by stressful events at work/school	No	234	39.3	92	39.8	142	39.0
	Yes	275	46.2	107	46.3	168	46.2
	Missing*	86	14.5	32	13.9	54	14.8
Affected by financial difficulties	No	216	36.3	82	35.5	134	36.8
	Yes	320	53.8	125	54.1	195	53.6
	Missing*	59	9.9	24	10.4	35	9.6
Affected by thoughts of previous stressful events	No	264	44.4	117	50.6	147	40.4
	Yes	284	47.7	97	42.0	187	51.4
	Missing*	47	7.9	17	7.4	30	8.2
**Migration-related characteristics**							
Year of migration	Year<1996	350	58.8	136	58.9	214	58.8
	Year≥1996	241	40.5	92	39.8	149	40.9
	Missing*	4	0.7	3	1.3	1	0.3
Ethnicity of friends in Germany	Resettlers	522	87.7	208	90.0	355	97.5
	Germans and other migrants	61	10.3	20	8.7	41	11.3
	Missing*	12	2.0	3	1.3	9	2.5
German language skills	Less than average	165	27.7	85	36.8	80	22.0
	Above average	428	71.9	145	62.8	283	77.7
	Missing*	2	0.3	1	0.4	1	0.3
**Health behaviour**							
Alcohol consumption	Current consumption	438	73.6	185	80.1	253	69.5
	Former consumption	60	10.1	27	11.7	33	9.1
	Never	63	10.6	7	3.0	56	15.4
	Missing*	34	5.7	12	5.2	22	6.0
Smoking behaviour	Current smoker	91	15.3	64	27.7	27	7.4
	Former smoker	116	19.5	88	38.1	28	7.7
	Never smoker	376	63.2	76	32.9	300	82.4
	Missing*	12	2.0	3	1.3	9	2.5
Moderate to vigorous physical activity	>2 hours/week	71	11.9	28	12.1	43	11.8
2 hours/week	64	10.8	30	13.0	34	9.3
<1 hour/week	114	19.2	29	12.6	85	23.4
No sports	323	54.3	132	57.1	191	52.5
	Missing*	23	3.9	12	5.2	11	3.0
Leisure-time physical activity	>2 hours/week	231	38.8	97	42.0	134	36.8
2 hours/week	137	23.0	52	22.5	85	23.4
<1 hour/week	119	20.0	35	15.2	84	23.1
No sports	102	17.1	45	19.5	57	15.7
	Missing*	6	1.0	2	0.9	4	1.1
Work-related physical activity	Vigorous	132	22.2	65	28.1	67	18.4
Moderate	186	31.3	82	35.5	104	28.6
Light	96	16.1	24	10.4	72	19.8
No physical work	110	18.5	33	14.3	77	21.2
	Missing*	71	11.9	27	11.7	44	12.1

* Number of missing in relation to Nn=595 (not part of the categories).

All participants socialised more among their ethnic group than with other migrants and natives. Most participants reported alcohol consumption, while there were more former tobacco smokers than current smokers.

Considering physical activity, MVPA was the least popular activity participated in by the Resettlers, with 54.3% reporting no activity and 11.9% reporting moderate–vigorous physical activity for more than 2 hours per week. About 38.8% of Resettlers reported LPA for more than 2 hours per week. The proportion of men and women who reported participating in physically tasking WPA was comparable, but double the number of women compared with men who reported light/no physically tasking WPA. The mean value of the EPA score was 2.6±1.8 both for men and women, TPA 4.3±2.1 for men and 3.9±2.1 for women and for perceived control 30.1±5.8 for men and 28.1±6.2 for women.

Regarding the multivariable models in table 3, the measure R² suggests a better model fit for men’s physical activity levels than that for women, with the WPA model for men’s activity achieving the best fit (R²=37%), and for women (R²=23%).

**Table 3:** Multivariable linear regression models, depicting the association between the increase in physical activity and independent predictors

		Total physical activity				Extracurricular physical activity				Work-related physical activity			
		**Men**		**Women**		**Men**		**Women**		**Men**		**Women**	
**Variable**		**B (95% CI)**	**P value**	**B (95% CI)**	**P value**	**B (95% CI)**	**P value**	**B (95% CI)**	**P value**	**B (95% CI)**	**P value**	**B (95% CI)**	**P value**
	Constant	1.56(−2.74 to 5.86)	0.474	−1.19(−5.59 to 3.21)	0.595	2.29(−1.47 to 6.06)	0.230	103.68(5.27 to 202.09)	0.039	−2.38(−3.86 to −0.89)	0.002	−3.32(−5.56 to −1.08)	0.004
**Demographics**													
Age		0.02(−0.01 to 0.06)	0.170	0.00(−0.03 to 0.03)	0.978	0.00(−0.02 to 0.03)	0.747	−0.02(−0.04 to 0.00)	0.044	0.02(0.00 to 0.03)	0.023	0.03(0.02 to 0.04)	<0.001
Educational level	University	Reference		Reference		Reference		Reference		Reference		Reference	
	Upper secondary school	−0.69(−1.78 to 0.40)	0.211	−0.41(−1.14 to 0.32)	0.268	−1.42(−2.39 to −0.46)	0.004	−0.52(−1.12 to 0.07)	0.085	0.84(0.36 to 1.31)	0.001	0.29(−0.09 to 0.66)	0.136
	Below upper secondary school	−0.26(−1.16 to 0.64)	0.566	0.09(−0.53 to 0.72)	0.771	−0.79(−1.61 to 0.02)	0.057	−0.23(−0.74 to 0.28)	0.371	0.53(0.13 to 0.93)	0.010	0.47(0.14 to 0.79)	0.005
Employment status	Unemployed	Reference		Reference						Reference		Reference	
	Employed	1.35(0.31 to 2.39)	0.012	1.21(0.51 to 1.91)	0.001					1.18(0.70 to 1.67)	<0.001	1.13(0.77 to 1.50)	<0.001
Marital status	Single			Reference				Reference					
	Divorced/ widowed			0.66(−0.01 to 1.33)	0.054			1.61(0.60 to 2.61)	0.002				
	Married							0.94(0.03 to 1.85)	0.042				
**Physiologicalcharacteristics**													
	Continuous BMI	−0.08(−0.17 to 0.01)	0.088			−0.06(−0.14 to 0.01)	0.099						
Frequent pain	No							Reference		Reference		Reference	
	Yes							−0.56(−1.01 to −0.11)	0.014	0.66(0.25 to 1.07)	0.002	0.29(0.01 to 0.58)	0.044
Diabetes	Yes	Reference				Reference							
	No	1.51(−0.14 to 3.17)	0.073			1.41(0.02 to 2.81)	0.047						
Tumour	Yes					Reference		Reference					
	No					1.59(0.18 to 3.01)	0.028	−1.11(−2.15 to −0.06)	0.038				
Myocardial infarction	Yes			Reference				Reference				Reference	
	No			4.14(0.32 to 7.95)	0.034			2.76(−0.36 to 5.87)	0.082			1.77(−0.20 to 3.74)	0.079
**Psychological stressors**													
Perceived control		0.07(0.01 to 0.14)	0.024							0.05(0.02 to 0.08)	0.003		
Affected by financial difficulties	No	Reference		Reference		Reference		Reference		Reference		Reference	
	Yes	−0.75(−1.50 to 0.00)	0.050			−0.99(−1.61 to −0.37)	0.002					0.38(0.10 to 0.66)	0.008
Feeling at home	No	Reference		Reference		Reference		Reference		Reference			
	Mostly							−0.67(−1.10 to −0.24)	0.003				
	Yes			0.47(−0.05 to 0.99)	0.075					−0.46(−0.80 to −0.12)	0.008		
**Migration-related characteristics**													
Year of migration								−0.05(−0.10 to 0.00)	0.042				
Ethnicity of friends in Germany	Germans and other migrants									Reference			
	Resettlers									0.59(−0.01 to 1.19)	0.055		
**Healthbehaviour**													
Smoking behaviour	Current Smoker			Reference		Reference							
	Former smoker			−0.87(−1.83 to 0.10)	0.078	0.86(0.11 to 1.62)	0.025						
	Never smoker					0.78(−0.07 to 1.63)	0.073						
**R²**		0.23		0.13		0.29		0.18		0.37		0.23	

The B reflects an increase in total physical activity

B, unstandardised regression coefficient

### Demographics

Increasing age was significantly associated with lower EPA in women, whereas in WPA, this was associated with increasing levels for both men and women.

Employed people reported more TPA and WPA. University-level educated men reported higher EPA. In contrast, both university-educated men and women reported significantly lower WPA in comparison to lower education levels. Marital status significantly influenced women’s EPA, with divorced and married women reporting more EPA than those who are single. Divorced/widowed and married women’s EPA increased by 1.61 and 0.94 hours/week compared with single women.

### Physiological characteristics

Both men’s and women’s TPA and EPA were positively associated with not having been diagnosed with a health condition or reported pain, but women who reported having a tumour were more likely to report higher levels of EPA. However, pain was associated with increasing levels of WPA in both men and women.

### Psychological stressors

Financial difficulties were significantly associated with women’s increasing WPA and decreasing men’s TPA and EPA. Perceived control was only associated with men’s TPA and WPA (online supplemental tables 6 and 7 further show the correlation of TPA and perceived control and the score distribution using a histogram among men and women, respectively). An increase in control of external stressors significantly increased men’s physical activity behaviour. Women who did not feel at home reported high levels of EPA than those mostly feeling at home; however, men who reported feeling at home reported significantly less physically tasking WPA by 0.46 hours/week in comparison to those who reported not feeling at home.

### Migration-related characteristics

The year of migration contributed significantly to women’s EPA, with women who migrated in recent years reporting less EPA by 0.05 hours/week.

### Health behaviour

Smoking behaviour was significantly associated with decreasing men’s EPA. To elucidate, men who formerly smoked reported 0.87 hours/week more EPA than those who smoked.

## Discussion

### Summary

To the best of our knowledge, this paper is the first to report on patterns and determinants of physical activity among ethnic German Resettlers from FSU in Germany. Findings confirm that physical activity is associated with age, employment, education, marital status, psychological stressors, migration background and health behaviour. Additionally, predictors of physical activity vary with sex based on sex and gender-related factors.^30^ For instance, in our study, more men report myocardial infarctions than women, whereas more women report more diabetes than men; however, we find that myocardial infarctions and diabetes are significant predictors of women’s and men’s physical activity, respectively, which could be linked to the higher risk of diabetic women compared with diabetic men developing heart failure.^31^ Additionally, perceived control over stressors was associated only with men’s physical activity. Previous research suggests that male Resettlers have particularly struggled with integration, psychological mastery and coherence, unlike women, and their role as family breadwinners was challenged by unemployment.^32 33^ On the other hand, unlike men, women’s EPA correlated to marital status, connecting the association with women’s roles in the home, seeing that EPA included household chores and activities, revealing an imprint of cultural roles in migrant homes.^34^ Some of these factors also influence differences in physical activity participation in the German population.^35^

In addition, migrants are diverse regarding their opportunities, socioeconomic status, linguistics, healthcare needs and migration reasons. Their ability to adopt healthy levels of physical activity is correlated to these ‘special’ influencing factors that may not impact the non-immigrant population.^36^ These ‘special’ factors shape health-related behaviours and exert distress, resulting in the reduction of perceived control, thus owing to poor health choices, diminished physical health, suicide and difficulties integrating that drive health inequity among migrants.^22 33 36^ Nevertheless, all types of physical activity can reverse the effects of distress, thereby increasing perceived control.^37^,^40^

### Results in reflection to literature

#### Demographics

Demographic patterns (age, education, employment and marital status) predict associations with physical activity similar to those of autochthonous communities.^35^ An increase in age, which often comes with the opportunity of employment up to a certain age, and higher education status are associated with higher physical activity. This is also exhibited in populations in Russia and the USA.^41 42^ As such, employment compared with unemployment among Resettlers owes to higher WPA due in part to opportunities to finance extracurricular activities^42^,^45^ and the workplace as the foundation of their daily life, which includes physical activity and social interaction.^46^ Notably, though active people are of a higher socioeconomic status, lower socioeconomic classes who may also report financial difficulties report higher WPA.^42^,^4547^ In 2009, male German Resettlers from the FSU had a lower adjusted net income (€1155) compared with other migrant groups and the native population, and a recent analysis identified that migrants earn much less than Germans in firms consisting of both migrants and Germans.^33 48 49^ Additionally, marital status was a significant predictor only for women’s EPA. Separation from a spouse due to divorce/death and marriage being emotionally intense transitions leading to women’s role changes in society are associated with increased EPA compared with single women. EPA was characterised by leisure activities that included household chores/hobbies, which a cultural imprint may have influenced. This could also elucidate why education level, unlike men’s, was not a significant predictor for women’s EPA. Literature shows that cultural imprints around gender influence migrant households and health.^50^ For instance, gender norms influence physical activity choices, with women focusing on familial and feminine activities and men on strength-related activities, a pattern identified in Russian-speaking Slavic immigrants in the USA.^50 51^ However, the associations between marital status/marital transitions and physical activity are inconclusive.^52^

#### Physiological characteristics

Sex differences have been identified in the association between health conditions and physical activity. Results portray a significant relationship between the associations of diabetes in men and myocardial infarction in women. Findings from a meta-analysis on diabetic populations showed that women have a higher risk of heart failure in comparison to men.^31^ Additionally, pain is linked to increased WPA in both men and women, likely due to WPA intensity. Despite having higher education, later-wave Resettlers in Germany often end up in lower-paying, intense, hazardous jobs because of poor language skills.^33 48^ Pain and other health conditions, such as CVD risk factors and site-specific cancer incidences, characterised more among Resettlers than the German population, cause physiological distress and thereby predict lower physical activity in our sample.^5 6^ Such conditions influence the perception of physical barriers, causing fear-avoidance behaviour, reduced endurance and reduced physical activity.^53^,^55^ This pattern is also observed in older Russian-speaking Slavic immigrants in the USA, where chronic conditions and age-related pain decrease physical activity.^51^ However, these immigrants recognise that physical activity manages deteriorating health and pain, reducing medication needs and improving functionality, which motivates continued physical activity.^51^ This is a key component in rehabilitation plans for cancer treatment, aiding in daily functioning and potentially improving tumour prognosis.^56 57^ Hence, this could have been a critical physical activity motivator in our sample among the women. It has been reported that more women than men accept rehabilitation treatment^58^ and regardless of the efforts and guidelines put in place to promote physical activity through rehabilitation services, men’s uptake and physical activity levels are low.^59^ Cancers and tumours can impact men’s mental well-being and challenge their sense of masculinity. Some qualitative studies suggest that embracing rehabilitation services may be viewed as reducing masculinity and eliciting feelings of sympathy and dependency, thus impeding independence.^59^,^61^

Irrespectively, there is limited research on the relationship between tumours and physical activity, particularly postdiagnosis.^62^ Nonetheless, the older Russian-speaking Slavic immigrants expressed a need for health education and practical applications of physical activity knowledge to enhance their well-being.^51^

#### Migration-related characteristics and psychological stressors

The uniqueness of the Resettlers compared with the autochthonous communities is encapsulated in their migration experience and its effects. Resettlers’ past experiences influence cognitive reconstructions, impacting physical activity.^25^ For instance, unlike women, perceived control is essential in predicting men’s physical activity. Notwithstanding, women mainly feeling (not entirely) at home equally report lower levels of EPA. Migrants, over their lifetime and generations, present with psychological distress based on their experiences, which can be attributed to Germans having long questioned their identity and affiliation, referring to them as Russians.^5 63^ Moreover, being a migrant can be a risk factor for suicide and reduced physical activity due in part to postmigration stress, socioeconomic deprivation, unemployment, poor integration and hate speech, which have all been experienced by Resettlers affecting men more than women due to stereotypes on masculinities, for example, men viewed as providers.^25 32 33 35 36^ Although more women than men across populations suffer from depressive disorders, depression is a significant disease burden in the male population and a cause of suicide^64^,^67^ that results in higher rates of death in men compared with women.^68^,^70^ The association of psychological health and physical activity is mainly bidirectional, and positive emotions predict increased physical activity.^40 71^ On the other hand, men who feel at home report lower intense WPA. This may partly reflect memory research findings showing that the meaning of past experiences can change based on current experiences.^25^ To elucidate, this can be attributed to job market integration, as earlier mentioned, leading to a lower likelihood of reporting high intense WPA, thereby making the duration of stay an essential predictor of physical activity. Likewise, a narrative synthesis in a systematic and meta-analysis review revealed that health risk factors, such as physical activity, increase with the duration of stay among migrants. However, results from the meta-analysis revealed lower odds of physical activity with increased duration of stay among migrants compared with natives. These inconsistencies have been credited to errors caused by self-reported physical activity.^72 73^ Regarding WPA, sex differences have been identified in the positive associations of WPA with ‘financial difficulties’ for women and ‘ethnicity of friends’ for men. This can be explained by results examined from a meta-analysis on gender/sex differences in the theory of mind and their effect on employment, where primary motivators are intrinsic (need for money) for women, unlike extrinsic factors (competition) for men. However, other literature portrays otherwise.^74 75^ Furthermore, an explanation of the associations between Resettler’s ethnicity of friends and physical activity can also be attached to the effect of networking on job leads, a phenomenon experienced across populations.^76^

#### Health behaviour

There is a sex difference in the significance of smoking on physical activity. Resettler men who stopped smoking report more EPA compared with those who are still smoking. These results may reflect a web of causations as social norms on acceptance of female smoking behaviour^77^ and the positive effect physical activity has in improving withdrawal intentions and symptoms and reducing tobacco cravings.^77^ Alongside, motivations for physical activity, such as physical activity endurance, disease prevention and physical appearance, oppose smoking effects.^77^,^80^

### Implications of result

While physical activity is known to be generally low among migrant populations, including Resettlers’, our results link associations with this phenomenon to different biological, psychosocial and behavioural factors. Similarities have been identified mainly between Resettlers and other migrants, including ethnic populations, but additional layers of biographies influenced by migration have been revealed among migrants. This outlines migration backgrounds as a distinction between migrants and ethnic populations. Embedded in the migrant background are various contextual factors adding to the complexity of physical activity behaviour, thus highlighting the need for a biopsychosocial perspective in physical activity research, a viewpoint that has also been advocated for by the WHO.^26 81^ Furthermore, our findings on the biopsychosocial associations probe for further transdisciplinary research on the interaction effects of the different levels of health investigated and their associations with physical activity. Hence, our findings are critical for programme implementers and policy actors in the physical activity space since they must account for variations in the biopsychosocial characteristics of their populations as they design programmes to combat non-communicable disease burdens through physical activity to achieve better outcomes.

### Methodological limitations

The main strengths of the study were that, due to extensive measures taken in the recruitment process, including a bilingual questionnaire, we could survey the largest sample of Resettlers. However, we suspect our sample may not have been representative because we did not survey many uneducated Resettlers.^5^

Moreover, the study assessed various domains of physical activity subjectively. This was because the questionnaire was designed to primarily assess the health status of the Resettlers, with a secondary focus on physical activity as a risk factor. To avoid dropouts due to a lengthy questionnaire, we had to make a trade-off between the length and completeness, including comprehensiveness. This has limitations because over-reporting and under-reporting physical activity behaviour may compromise the tool’s reliability. Notwithstanding, our measure had strengths that objective measures could not accord in that it gave a contextual understanding of physical activity domains through examples of activities attached to each domain, thus capturing the Resettler’s cultural perceptions of physical activity to a degree. Also, regarding the mistrust many migrants have in authorities due to their past experiences in their home countries, wearing a device that would constantly record their activity could have been both burdensome and intrusive.^5 82^

During analysis, we also identified a variability in the models R² between the men and women. This variability could be due to various factors, for instance, due to the subjective assessment of physical activity, which was embedded in a comprehensive evaluation of the health status and, hence, might not have been detailed enough to decipher physical activity among female Resettlers in more detail. Additionally, the questionnaire might not have been sensitive enough to detect other factors that could contribute to the physical activity patterns among female Resettlers. Further research may be required, especially among female Resettlers, to examine the factors that impact physical activity. A qualitative approach could be valuable in identifying the enablers and barriers influencing women Resettler’s physical activity, a subject that has not yet been thoroughly explored in previous studies.

The main weakness in our analysis is missing data on a critical physical activity predictor (pain). The model’s inability to account for the predictor leads to variable bias, resulting in reduced statistical power and a possible misinterpretation of results. The missing data on ‘frequent pain’ could have been owed to uncertainty among the participants on what was meant by ‘frequent’. The assessment of pain was done superficially because we were interested in getting an overall picture of the health status of the Resettlers as a first step rather than already looking into details of specific associations.

## Conclusion

Some psychological stressors are unique to migrants, unlike the autochthonous population, due to their migration and integration experience, which in turn influence Resettlers’ psychological health.^36^ Although physical activity may reverse the effects of such stressors, thereby increasing the individual’s perceived control,^39 40^ stressors likewise pose as physical activity barriers.^40 71^ Perceived control over stressors was associated only with men’s TPA and WPA, emphasising the need to comprehend the root and association between health behaviour and psychological distress control among male Resettlers who are characterised by higher suicide rates, unlike women.

Furthermore, cultural imprints among the Resettlers may influence the essence of physical activity choice, frequency and intensity due to gender norms and social roles more so in familial homes. To comprehend the sex differences, researchers should seek to empirically assess why the sex variances occur to increase further the effectiveness of health-enhancing physical activity promotion programmes to all.

Physical activity among Resettlers can be used to improve integration, reduce psychological distress and increase physical functioning among those with disabling chronic conditions, thereby maintaining their role and needs in society. Our findings and literature show that health promotion programmes should target not only Resettlers but also other migrant communities burdened by chronic conditions due in part to physical inactivity to educate not only about healthy lifestyle choices but also their practical applications in life. While doing so, such programmes should also consider the impact of migrant biographies on health.

## supplementary material

10.1136/bmjopen-2024-086042 online supplemental file 1

## Data Availability

All data relevant to the study are included in the article or uploaded as supplementary information.

## References

[1] Statistisches Bundesamt (Destatis) (2020). Population with a Migrant Background up 2.1% in 2019: Lowest Increase since 2011.

[2] Westphal M (1999). Familiäre Und Berufliche Orientierungen von Aussiedlerinnen, in Aussiedler: Deutsche Einwanderer Aus Osteuropa.

[3] Panagiotidis J (2018). Emigrant. http://www.bpb.de/gesellschaft/migration/dossier-migration/247811/aussiedler?p=all.

[4] Aparicio ML, Döring A, Mielck A (2005). Differences between Eastern European immigrants of German origin and the rest of the German population in health status, health care use and health behaviour: a comparative study using data from the KORA-Survey 2000. Soz Praventivmed.

[5] Stolpe S, Ouma M, Winkler V (2018). Self-rated health among migrants from the former Soviet Union in Germany: a cross-sectional study. BMJ Open.

[6] Winkler V, Kaucher S, Deckert A (2019). Aussiedler Mortality (AMOR): cohort studies on ethnic German migrants from the Former Soviet Union. BMJ Open.

[7] Kaucher S, Leier V, Deckert A (2017). Time trends of cause-specific mortality among resettlers in Germany, 1990 through 2009. Eur J Epidemiol.

[8] Benziger CP, Roth GA, Moran AE (2016). The Global Burden of Disease Study and the Preventable Burden of NCD. Glob Heart.

[9] World Health Organisation (2017). Cardiovascular diseases (CVDS) [fact sheet].

[10] World Health Organisation (2020). The top 10 causes of death [fact sheet]. https://www.who.int/news-room/fact-sheets/detail/the-top-10-causes-of-death.

[11] World Health Organization (2018). ACTIVE: a technical package for increasing physical activity, Geneva.

[12] Kraus WE, Powell KE, Haskell WL (2019). Physical Activity, All-Cause and Cardiovascular Mortality, and Cardiovascular Disease. Med Sci Sports Exerc.

[13] World Health Organization (2022). Physical activity [fact sheet].

[14] Breda J, Jakovljevic J, Rathmes G (2018). Promoting health-enhancing physical activity in Europe: Current state of surveillance, policy development and implementation. Health Policy.

[15] World Health Organisation (2018). Germany physical activity [factscheet].

[16] Reimers AK, Brzoska P, Niessner C (2019). Are there disparities in different domains of physical activity between school-aged migrant and non-migrant children and adolescents? Insights from Germany. PLoS ONE.

[17] Schumann M, Kajikhina K, Polizzi A (2019). Concepts for migration-sensitive health monitoring. *J Health Monit*.

[18] Krist L, Dornquast C, Reinhold T (2020). Physical Activity Trajectories among Persons of Turkish Descent Living in Germany-A Cohort Study. Int J Environ Res Public Health.

[19] Biddle L, Hintermeier M, Costa D (2023). Context, health and migration: a systematic review of natural experiments. E Clin Med.

[20] Arena C, Holmberg C, Winkler V (2022). The Health Status and Healthcare Utilization of Ethnic Germans in Russia. IJERPH.

[21] Wieland ML, Tiedje K, Meiers SJ (2015). Perspectives on physical activity among immigrants and refugees to a small urban community in Minnesota. J Immigr Minor Health.

[22] Sandström E, Bolmsjö I, Janzon E (2015). Attitudes to and Experiences of Physical Activity among Migrant Women from Former Yugoslavia: -- a qualitative interview study about physical activity and its beneficial effect on heart health, in Malmö, Sweden. *AIMS Public Health*.

[23] Smith R, Spaaij R, McDonald B (2019). Migrant Integration and Cultural Capital in the Context of Sport and Physical Activity: a Systematic Review. Int Migration & Integration.

[24] Caron RM, Rodrigues Amorim Adegboye A, Moreno-Leguizamon CJ (2022). Editorial: The Impact of Migration and Resettlement on Health. Front Public Health.

[25] Thiel A, Sudeck G, Gropper H (2020). The iReAct study - A biopsychosocial analysis of the individual response to physical activity. Contemp Clin Trials Commun.

[26] John JM, Haug V, Thiel A (2020). Physical Activity Behavior from a Transdisciplinary Biopsychosocial Perspective: a Scoping Review. Sports Med Open.

[27] Centers for Disease Control and Prevention (2023). How to measure physical activity intensity. https://www.cdc.gov/physical-activity-basics/measuring/index.html.

[28] Ajzen I (1991). The theory of planned behavior. Organ Behav Hum Decis Process.

[29] (2010). A healthy lifestyle - WHO recommendations [fact sheet]. https://www.who.int/europe/news-room/fact-sheets/item/a-healthy-lifestyle---who-recommendations.

[30] Halliday AJ, Kern ML, Turnbull DA (2019). Can physical activity help explain the gender gap in adolescent mental health? A cross-sectional exploration. Ment Health Phys Act.

[31] Ohkuma T, Komorita Y, Peters SAE (2019). Diabetes as a risk factor for heart failure in women and men: a systematic review and meta-analysis of 47 cohorts including 12 million individuals. Diabetologia.

[32] Kyobutungi C, Ronellenfitsch U, Razum O (2006). Mortality from external causes among ethnic German immigrants from former Soviet Union countries, in Germany. Eur J Public Health.

[33] Deckert A, Winkler V, Meisinger C (2015). Suicide and external mortality pattern in a cohort of migrants from the former Soviet Union to Germany. J Psychiatr Res.

[34] Doblhammer G, Gumà J (2018). A Demographic Perspective on Gender, Family and Health in Europe.

[35] Abu-Omar K, Messing S, Sarshar M (2021). Sociodemographic correlates of physical activity and sport among adults in Germany: 1997–2018. *Ger J Exerc Sport Res*.

[36] Kurth B-M, Razum O (2019). Editorial: Health monitoring should reflect population diversity. J Health Monit.

[37] Bergouignan A, Legget KT, De Jong N (2016). Effect of frequent interruptions of prolonged sitting on self-perceived levels of energy, mood, food cravings and cognitive function. Int J Behav Nutr Phys Act.

[38] Lindberg CM, Srinivasan K, Gilligan B (2018). Effects of office workstation type on physical activity and stress. Occup Environ Med.

[39] Paolucci EM, Loukov D, Bowdish DME (2018). Exercise reduces depression and inflammation but intensity matters. Biol Psychol.

[40] Schultchen D, Reichenberger J, Mittl T (2019). Bidirectional relationship of stress and affect with physical activity and healthy eating. Br J Health Psychol.

[41] Kolosnitsyna M, Khorkina N, Lopatina M (2018). The Factors of Physical Activities in Russian Youth: Evidence from Micro-Data. *SSRN Journal*.

[42] Scholes S, Bann D (2018). Education-related disparities in reported physical activity during leisure-time, active transportation, and work among US adults: repeated cross-sectional analysis from the National Health and Nutrition Examination Surveys, 2007 to 2016. BMC Public Health.

[43] Stalsberg R, Pedersen AV (2018). Are Differences in Physical Activity across Socioeconomic Groups Associated with Choice of Physical Activity Variables to Report?. Int J Environ Res Public Health.

[44] Buková A, Chovanová E, Küchelová Z (2021). Association between Educational Level and Physical Activity in Chronic Disease Patients of Eastern Slovakia. Healthcare (Basel).

[45] Huikari S, Junttila H, Ala-Mursula L (2021). Leisure-time physical activity is associated with socio-economic status beyond income – Cross-sectional survey of the Northern Finland Birth Cohort 1966 study. Econ Hum Biol.

[46] Chaudhury H, Campo M, Michael Y (2016). Neighbourhood environment and physical activity in older adults. Soc Sci Med.

[47] Williams A, Nolan TS, Luthy J (2024). Association of Socioeconomic Status With Life’s Essential 8 in the National Health and Nutrition Examination Survey: Effect Modification by Sex. J Am Heart Assoc.

[48] Bade KJ (2003). Aussiedler: Deutsche Einwanderer Aus Osteuropa.

[49] Brunow S, Jost O (2019). Wages of migrant and native employees in Germany: new light on an old issue. IAB-discussion paper.

[50] Wandschneider L, Batram-Zantvoort S, Razum O (2020). Representation of gender in migrant health studies - a systematic review of the social epidemiological literature. Int J Equity Health.

[51] Purath J, Van Son C, Corbett CF (2011). Physical activity: exploring views of older Russian-speaking slavic immigrants. Nurs Res Pract.

[52] Puciato D, Rozpara M (2021). Physical activity and socio-economic status of single and married urban adults: a cross-sectional study. PeerJ.

[53] Eronen J, von Bonsdorff MB, Törmäkangas T (2014). Barriers to outdoor physical activity and unmet physical activity need in older adults. Prev Med.

[54] Strohmeier F (2016). Barriers and their Influence on the Mobility Behavior of Elder Pedestrians in Urban Areas: Challenges and Best Practice for Walkability in the City of Vienna. Transp Res Procedia.

[55] Hasenbring MI, Verbunt JA (2010). Fear-avoidance and endurance-related responses to pain: new models of behavior and their consequences for clinical practice. Clin J Pain.

[56] American Society of Clinical Oncology (2019). Exercise during cancer treatment. https://www.cancer.net/survivorship/healthy-living/exercise-during-cancer-treatment.

[57] Büntzel J, Kusterer I, Rudolph Y (2017). Cancer Patients’ Knowledge and Acceptance of Physical Activities for Rehabilitation. In Vivo.

[58] Ohlsson-Nevo E, Alkebro I, Ahlgren J (2019). Cancer patients’ interest in participating in cancer rehabilitation. Acta Oncol.

[59] Rørth M, Tjørnhøj-Thomsen T, Cormie P (2019). Attitudes and Experiences of Men With Prostate Cancer on Risk in the Context of Injuries Related to Community-Based Football-A Qualitative Study. J Aging Phys Act.

[60] Andreasson J, Johansson T, Danemalm-Jägervall C (2023). Prostate Cancer and the Emotionology of Masculinity: Joking, Intellectualization, and the Reconstruction of Gender. J Men's Stud.

[61] Handberg C, Lomborg K, Nielsen CV (2015). Understanding male cancer patients’ barriers to participating in cancer rehabilitation. Eur J Cancer Care (Engl).

[62] Holmes MD, Chen WY, Feskanich D (2005). Physical activity and survival after breast cancer diagnosis. JAMA.

[63] Bade KJ, Oltmer J (1999). Aussiedler: Deutsche Einwanderer Aus Osteuropa.

[64] Seedat S, Scott KM, Angermeyer MC (2009). Cross-national associations between gender and mental disorders in the World Health Organization World Mental Health Surveys. Arch Gen Psychiatry.

[65] Charlson FJ, Baxter AJ, Dua T (2015). Excess mortality from mental, neurological and substance use disorders in the Global Burden of Disease Study 2010. Epidemiol Psychiatr Sci.

[66] Cavanagh JTO, Carson AJ, Sharpe M (2003). Psychological autopsy studies of suicide: a systematic review. Psychol Med.

[67] Turecki G, Brent DA (2016). Suicide and suicidal behaviour. Lancet.

[68] Canetto SS (2015). Suicide: Why Are Older Men So Vulnerable. Men Masc.

[69] Bostwick JM, Pabbati C, Geske JR (2016). Suicide Attempt as a Risk Factor for Completed Suicide: Even More Lethal Than We Knew. Am J Psychiatry.

[70] Vijayakumar L (2015). Suicide in women. Ind J Psychiatry.

[71] Jekauc D, Brand R (2017). Editorial: How do Emotions and Feelings Regulate Physical Activity?. Front Psychol.

[72] Jung M, Kim H, Loprinzi PD (2022). Association among length of residence, physical activity, and obesity in the US immigrants: A regression-based mediation analysis. Am J Hum Biol.

[73] Juárez SP, Honkaniemi H, Gustafsson N-K (2022). Health Risk Behaviours by Immigrants’ Duration of Residence: A Systematic Review and Meta-Analysis. Int J Public Health.

[74] Ridinger G, McBride M (2015). Money Affects Theory of Mind Differently by Gender. PLoS One.

[75] Štefko R, Bačík R, Fedorko R (2017). GENDER DIFFERENCES IN THE CASE OF WORK SATISFACTION AND MOTIVATION. PJMS.

[76] Chetty R, Jackson MO, Kuchler T (2022). Social capital I: measurement and associations with economic mobility. Nature New Biol.

[77] Tie Y, Tian W, Chen Y (2023). The relationship between physical exercise and smoking behavior among Chinese residents aged 16 years and older. Sci Rep.

[78] Rahman MM, Liang CY, Gu D (2019). Understanding Levels and Motivation of Physical Activity for Health Promotion among Chinese Middle-Aged and Older Adults: A Cross-Sectional Investigation. J Healthc Eng.

[79] Denche-Zamorano Á, Mendoza-Muñoz DM, Pereira-Payo D (2022). Does Physical Activity Reduce the Risk of Perceived Negative Health in the Smoking Population?. Int J Environ Res Public Health.

[80] Parmar MP, Kaur M, Bhavanam S (2023). A Systematic Review of the Effects of Smoking on the Cardiovascular System and General Health. Cureus.

[81] World Health Organization International Classification of Functioning, Disability, and Health: Children & Youth Version: ICF-CY.

[82] Sylvia LG, Bernstein EE, Hubbard JL (2014). Practical guide to measuring physical activity. J Acad Nutr Diet.

