# Supplementary material for: Biopsychosocial factors associated with physical activity among Resettlers of the former Soviet Union in Germany: a cross-sectional analysis
Source: BMJ Open. 2024 Dec 9;14(12):e086042. doi: 10.1136/bmjopen-2024-086042 (PMC11628956; doi:10.1136/bmjopen-2024-086042)

**Supplemental material**

Figure1: Study recruitment flow chart

**
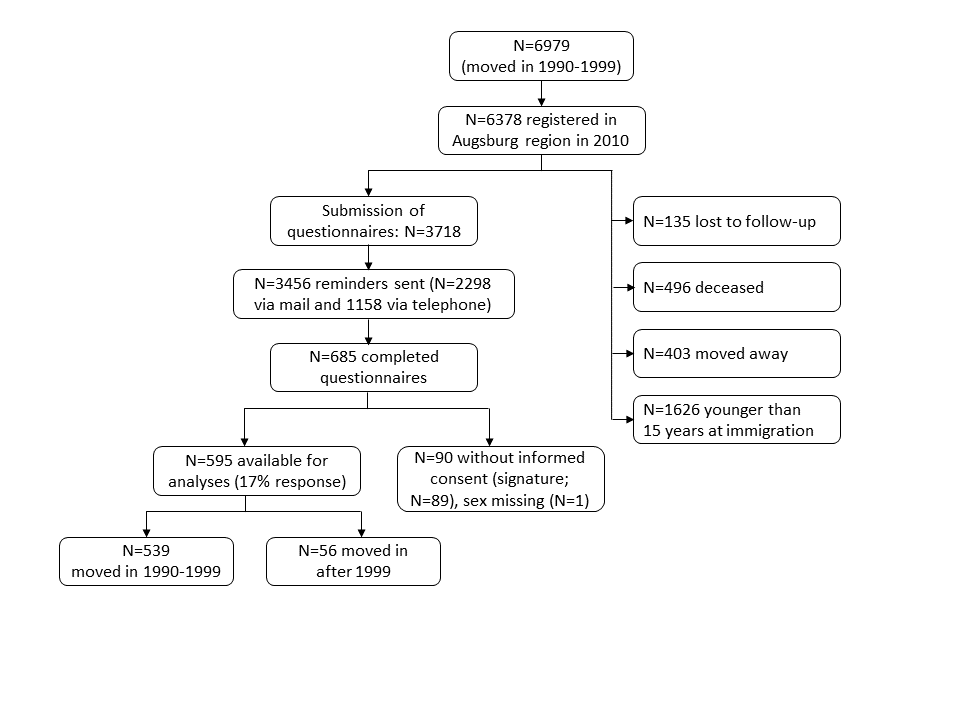
**

Table 1: Recodes of the 8 perceived behavioral control items.

|  | Never | Rarely | Sometimes | Often | Always |
| --- | --- | --- | --- | --- | --- |
| Feeling energetic | 1 | 2 | 3 | 4 | 5 |
| Feeling calm | 1 | 2 | 3 | 4 | 5 |
| Feeling Happy | 1 | 2 | 3 | 4 | 5 |
| Feeling worn-out | 5 | 4 | 3 | 2 | 1 |
| Feeling anxious | 5 | 4 | 3 | 2 | 1 |
| Feeling sad | 5 | 4 | 3 | 2 | 1 |
| Feeling doubtful | 5 | 4 | 3 | 2 | 1 |
| Feeling Lonely | 5 | 4 | 3 | 2 | 1 |

Table 2: Full models of the multivariable linear regression, depicting the association between the increase in physical activity and independent predictors

|  | **Variable** | **Total physical activity** | | | | **Extracurricular Physical Activity** | | | | **Work-related Physical Activity** | | | |
| --- | --- | --- | --- | --- | --- | --- | --- | --- | --- | --- | --- | --- | --- |
|  |  | **Men** | | **Women** | | **Men** | | **Women** | | **Men** | | **Women** | |
|  |  | **B (95% CI)** | **p** | **B (95% CI)** | **p** | **B (95% CI)** | **p** | **B (95% CI)** | **p** | **B (95% CI)** | **p** | **B (95% CI)** | **p** |
| Constant |  | 49.76(-104.19; 203.71) | 0.523 | 96.30(-43.66; 236.25) | 0.176 | 28.88(-102.52; 160.27) | 0.664 | 112.24(-2.52; 226.99) | 0.055 | 5.10(-66.75; 76.96) | 0.888 | -30.93(-104.91; 43.05) | 0.410 |
| **Demographics** |  |  |  |  |  |  |  |  |  |  |  |  |  |
| Age |  | 0.00(-0.04; 0.05) | 0.906 | 0.01(-0.02; 0.04) | 0.555 | -0.01(-0.05; 0.03) | 0.784 | -0.01(-0.04; 0.01) | 0.354 | 0.01(-0.01; 0.03) | 0.296 | 0.02(0.01; 0.04) | 0.005 |
| Educational level | University | Reference |  | Reference |  | Reference |  | Reference |  | Reference |  | Reference |  |
|  | Upper secondary school | -0.56(-1.79; 0.66) | 0.363 | -0.24(-1.01; 0.54) | 0.545 | -1.28(-2.32; -0.23) | 0.017 | -0.49(-1.13; 0.14) | 0.127 | 0.65(0.08; 1.21) | 0.025 | 0.28(-0.12; 0.68) | 0.166 |
|  | Below upper sec. school | -0.46(-1.54; 0.63) | 0.408 | 0.18(-0.49; 0.86) | 0.597 | -0.90(-1.83; 0.03) | 0.056 | -0.19(-0.75; 0.36) | 0.496 | 0.34(-0.17; 0.86) | 0.189 | 0.42(0.07; 0.77) | 0.019 |
| Employment status | Unemployed | Reference |  | Reference |  | Reference |  | Reference |  | Reference |  | Reference |  |
|  | Employed | 1.45(0.09; 2.81) | 0.036 | 1.03(0.19; 1.87) | 0.017 | -0.01(-1.17; 1.15) | 0.989 | -0.02(-0.71; 0.67) | 0.959 | 1.19(0.52; 1.85) | 0.001 | 0.85(0.41; 1.30) | 0.000 |
| Marital status | Single | Reference |  | Reference |  | Reference |  | Reference |  | Reference |  | Reference |  |
|  | Divorced/ widowed | 1.15(-1.34; 3.64) | 0.363 | 1.70(0.40; 3.01) | 0.011 | 1.00(-1.12; 3.13) | 0.351 | 1.83(0.75; 2.90) | 0.001 | 0.58(-0.61; 1.77) | 0.337 | -0.14(-0.84; 0.56) | 0.692 |
|  | Married | 1.61(-0.46; 3.68) | 0.125 | 1.00(-0.18; 2.18) | 0.095 | 1.23(-0.54; 2.99) | 0.171 | 1.07(0.11; 2.04) | 0.030 | 0.54(-0.39; 1.47) | 0.249 | -0.13(-0.76; 0.50) | 0.684 |
| **Physiological Characteristics** |  |  |  |  |  |  |  |  |  |  |  |  |  |
|  | BMI | -0.09(-0.19; 0.01) | 0.075 | -0.02(-0.07; 0.04) | 0.549 | -0.08(-0.17; 0.01) | 0.070 | -0.03(-0.07; 0.02) | 0.211 | -0.01(-0.06; 0.04) | 0.587 | 0.02(-0.01; 0.04) | 0.262 |
| Frequent pain | No | Reference |  | Reference |  | Reference |  | Reference |  | Reference |  | Reference |  |
|  | Yes | 0.78(-0.27; 1.83) | 0.142 | -0.02(-0.78; 0.74) | 0.960 | 0.27(-0.63; 1.16) | 0.554 | -0.34(-0.97; 0.28) | 0.283 | 0.59(0.10; 1.07) | 0.018 | 0.33(-0.07; 0.74) | 0.108 |
| Diabetes | Yes | Reference |  | Reference |  | Reference |  | Reference |  | Reference |  | Reference |  |
|  | No | 2.22(0.35; 4.09) | 0.020 | 0.00(-1.00; 1.01) | 0.995 | 1.48(-0.11; 3.08) | 0.068 | -0.10(-0.93; 0.72) | 0.809 | 0.73(-0.29; 1.75) | 0.160 | 0.04(-0.51; 0.59) | 0.881 |
| Tumor | Yes | Reference |  | Reference |  | Reference |  | Reference |  | Reference |  | Reference |  |
|  | No | 1.05(-0.80; 2.90) | 0.262 | -1.20(-2.59; 0.19) | 0.089 | 1.44(-0.14; 3.02) | 0.073 | -1.14(-2.28; 0.00) | 0.049 | -0.15(-0.98; 0.68) | 0.722 | -0.13(-0.88; 0.61) | 0.723 |
|  |  |  |  |  |  |  |  |  |  |  |  |  |  |
| Myocardial infarction | Yes | Reference |  | Reference |  | Reference |  | Reference |  | Reference |  | Reference |  |
|  | No | -0.78(-2.53; 0.97) | 0.378 | 4.43(0.37; 8.48) | 0.033 | 0.11(-1.39; 1.60) | 0.888 | 2.84(-0.49; 6.17) | 0.094 | -0.70(-1.56; 0.16) | 0.111 | 1.89(-0.20; 3.98) | 0.076 |
|  |  |  |  |  |  |  |  |  |  |  |  |  |  |
| Stroke | Yes | Reference |  | Reference |  | Reference |  | Reference |  | Reference |  | Reference |  |
|  | No | -1.14(-3.40; 1.11) | 0.317 | 1.50(-1.47; 4.48) | 0.320 | -0.84(-2.77; 1.08) | 0.387 | 1.40(-1.04; 3.84) | 0.260 | -0.42(-1.55; 0.71) | 0.459 | 0.42(-1.12; 1.96) | 0.590 |
| Mobility Restrictions | No | Reference |  | Reference |  | Reference |  | Reference |  | Reference |  | Reference |  |
|  | Yes | -0.50(-1.42; 0.43) | 0.291 | -0.24(-0.92; 0.44) | 0.486 | -0.39(-1.18; 0.40) | 0.328 | -0.26(-0.82; 0.30) | 0.356 | -0.14(-0.56; 0.28) | 0.502 | 0.05(-0.30; 0.40) | 0.797 |
| **Psychological stressors** |  |  |  |  |  |  |  |  |  |  |  |  |  |
| Perceived control |  | 0.07(-0.02; 0.17) | 0.119 | 0.02(-0.04; 0.09) | 0.527 | 0.03(-0.05; 0.11) | 0.394 | 0.01(-0.05; 0.06) | 0.763 | 0.03(-0.01; 0.07) | 0.142 | 0.01(-0.02; 0.04) | 0.548 |
| Affected by family difficulties | No | Reference |  | Reference |  | Reference |  | Reference |  | Reference |  | Reference |  |
|  | Yes | -0.85(-1.81; 0.10) | 0.079 | -0.03(-0.67; 0.61) | 0.924 | -0.53(-1.34; 0.29) | 0.202 | 0.12(-0.40; 0.65) | 0.640 | -0.34(-0.77; 0.08) | 0.112 | -0.23(-0.56; 0.10) | 0.167 |
| Affected by stressful events at work/school | No | Reference |  | Reference |  | Reference |  | Reference |  | Reference |  | Reference |  |
|  | Yes | -0.05(-1.02; 0.92) | 0.919 | 0.17(-0.50; 0.84) | 0.612 | 0.04(-0.79; 0.87) | 0.927 | -0.09(-0.64; 0.46) | 0.739 | -0.07(-0.49; 0.36) | 0.758 | 0.27(-0.07; 0.62) | 0.122 |
| Affected by financial difficulties | No | Reference |  | Reference |  | Reference |  | Reference |  | Reference |  | Reference |  |
|  | Yes | -0.93(-1.75; -0.11) | 0.027 | 0.20(-0.42; 0.82) | 0.523 | -0.87(-1.57; -0.17) | 0.015 | -0.04(-0.55; 0.47) | 0.885 | -0.09(-0.47; 0.29) | 0.640 | 0.29(-0.03; 0.61) | 0.077 |
| Affected by thoughts of previous stressful events | No | Reference |  | Reference |  | Reference |  | Reference |  | Reference |  | Reference |  |
|  | Yes | 0.28(-0.51; 1.08) | 0.481 | 0.01(-0.62; 0.63) | 0.979 | 0.23(-0.45; 0.90) | 0.510 | -0.16(-0.67; 0.35) | 0.533 | 0.00(-0.37; 0.37) | 0.995 | 0.12(-0.21; 0.45) | 0.473 |
| Feeling at home | No | Reference |  | Reference |  | Reference |  | Reference |  | Reference |  | Reference |  |
|  | Mostly | -0.95(-2.13; 0.22) | 0.110 | -0.25(-1.41; 0.91) | 0.671 | -0.52(-1.53; 0.48) | 0.302 | -0.76(-1.71; 0.19) | 0.118 | -0.30(-0.84; 0.24) | 0.278 | 0.56(-0.06; 1.19) | 0.078 |
|  | Yes | -0.80(-2.01; 0.41) | 0.191 | 0.18(-1.07; 1.43) | 0.779 | -0.08(-1.11; 0.95) | 0.873 | -0.25(-1.28; 0.78) | 0.629 | -0.64(-1.18; -0.10) | 0.021 | 0.46(-0.22; 1.14) | 0.183 |
| **Migration-related characteristics** |  |  |  |  |  |  |  |  |  |  |  |  |  |
| Year of migration |  | -0.02(-0.10; 0.05) | 0.535 | -0.05(-0.12; 0.02) | 0.162 | -0.01(-0.08; 0.05) | 0.683 | -0.06(-0.11; 0.00) | 0.056 | 0.00(-0.04; 0.03) | 0.877 | 0.01(-0.02; 0.05) | 0.476 |
| Ethnicity of friends in Germany | Germans and other migrants | Reference |  | Reference |  | Reference |  | Reference |  | Reference |  | Reference |  |
|  | Resettlers | 0.61(-0.89; 2.11) | 0.421 | 0.10(-0.79; 0.99) | 0.819 | -0.01(-1.29; 1.27) | 0.988 | -0.01(-0.74; 0.72) | 0.986 | 0.76(0.05; 1.48) | 0.036 | 0.18(-0.28; 0.64) | 0.434 |
| German language skills | Less than average | Reference |  | Reference |  | Reference |  | Reference |  | Reference |  | Reference |  |
|  | Above average | 0.08(-0.84; 1.00) | 0.865 | 0.12(-0.65; 0.89) | 0.760 | 0.38(-0.40; 1.17) | 0.336 | 0.18(-0.46; 0.81) | 0.582 | -0.21(-0.63; 0.21) | 0.329 | 0.02(-0.38; 0.43) | 0.903 |
| **Health behavior** |  |  |  |  |  |  |  |  |  |  |  |  |  |
| Alcohol consumption | current consumption | Reference |  | Reference |  | Reference |  | Reference |  | Reference |  | Reference |  |
|  | Former consumption | 0.76(-0.60; 2.13) | 0.271 | -0.39(-1.53; 0.75) | 0.500 | 0.76(-0.40; 1.93) | 0.196 | -0.13(-1.07; 0.80) | 0.781 | -0.04(-0.69; 0.61) | 0.905 | -0.17(-0.78; 0.44) | 0.582 |
|  | Never | -1.96(-6.69; 2.77) | 0.413 | -0.70(-1.58; 0.19) | 0.123 | -1.73(-5.77; 2.31) | 0.397 | -0.59(-1.31; 0.14) | 0.113 | -0.15(-2.24; 1.95) | 0.890 | -0.19(-0.65; 0.26) | 0.402 |
| Smoking behavior | Current Smoker | Reference |  | Reference |  | Reference |  | Reference |  | Reference |  | Reference |  |
|  | Former smoker | 0.70(-0.27; 1.68) | 0.156 | -0.85(-2.26; 0.56) | 0.237 | 0.94(0.10; 1.77) | 0.028 | -0.13(-1.28; 1.03) | 0.826 | -0.23(-0.67; 0.21) | 0.300 | -0.74(-1.48; -0.01) | 0.047 |
|  | Never smoker | 0.84(-0.23; 1.91) | 0.124 | -0.26(-1.35; 0.84) | 0.643 | 0.89(-0.03; 1.81) | 0.057 | 0.10(-0.80; 1.00) | 0.831 | -0.15(-0.64; 0.34) | 0.545 | -0.33(-0.89; 0.23) | 0.246 |
| **R²** |  | 0.35 |  | 0.19 |  | 0.38 |  | 0.22 |  | 0.44 |  | 0.30 |  |

Table 3: Total physical activity histogram


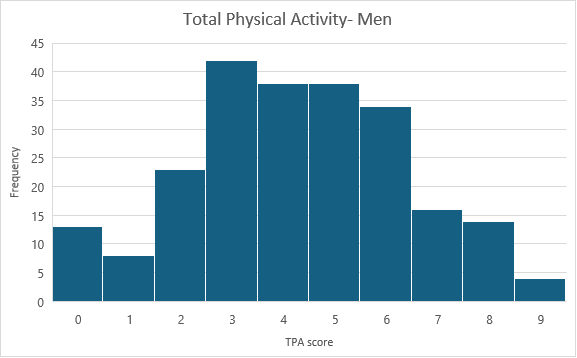


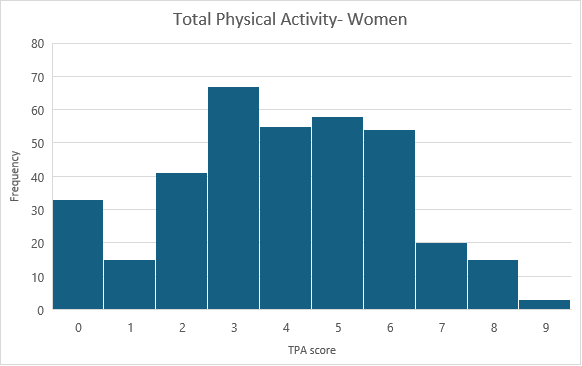


Table 4: Extracurricular physical activity histogram

Table 5: Work-related physical activity histogram

Table 6: Spearman correlation between total physical activity and perceived behavioral control stratified by sex

|  | Perceived behavioral control |
| --- | --- |
| **Men** |  |
| Total physical activity | 0.237^**^ |
| **Women** |  |
| Total physical activity | 0.08 |

** P< 0.01 (2-tailed). Men N= 196 Women N= 303

Table 7: Perceived behavioral control histogram
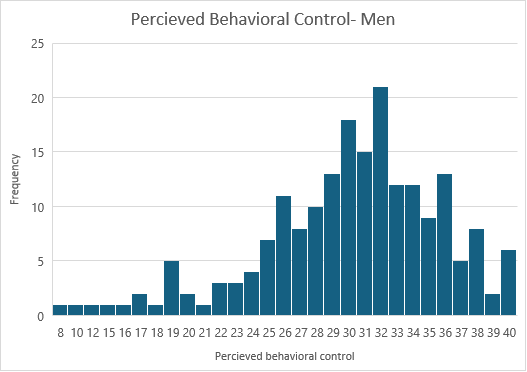


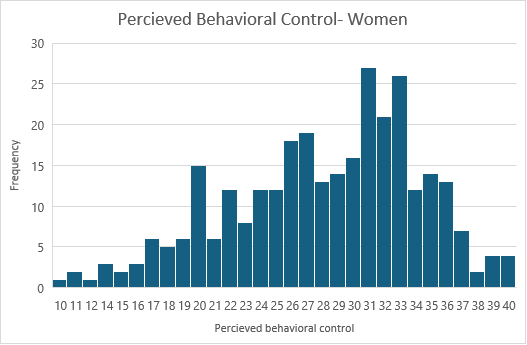

Supplement: online supplemental file 1 [file bmjopen-14-12-s001.docx]
