# Supplementary material for: Biopsychosocial factors associated with physical activity among Resettlers of the former Soviet Union in Germany: a cross-sectional analysis
Source: BMJ Open. 2024 Dec 9;14(12):e086042. doi: 10.1136/bmjopen-2024-086042 (PMC11628956; doi:10.1136/bmjopen-2024-086042)
Supplement: Supplementary file 2 [file bmjopen-2024-086042.reviewer_comments.pdf]

PEER REVIEW HISTORY

BMJ Open publishes all reviews undertaken for accepted manuscripts. Reviewers are asked to complete a checklist review form and are provided with free text boxes to elaborate on their assessment. These free text comments are reproduced below.

ARTICLE DETAILS

Title (Provisional)

A cross-sectional analysis of bio-psychosocial factors correlated with physical activity among Resettlers of the former Soviet Union in Germany

Authors

Ouma, Mary Achieng; Juma, Kenneth; Meisinger, Christa; Stolpe, Susanne; Becher, Heiko; Winkler, Volker Franz; Deckert, Andreas

---

VERSION 1 - REVIEW

**Reviewer**	**1**
**Name**	**Jung, Myungjin**
**Affiliation**	**University of North Texas, Kinesiology, Health Promotion, and Recreation**
**Date**	**12-Aug-2024**
**COI**	**None.**

August 12, 2024

A cross-sectional analysis of bio-psychological factors correlated with physical activity among female and male resettlers of the former Soviet Union

Decision: Major Revision

General comments: The manuscript is a valuable contribution to the understanding of physical activity among Resettlers in Germany, an often-overlooked population in health research. The study aims to investigate the relationship between physical activity and various bio-psychosocial factors, including sociodemographic, physiological, psychological, migration experience, and health behavior factors among male and female Resettlers from the Former Soviet Union in Germany. The authors conducted a cross-sectional study using a robust sample of 595 Resettlers, with data collected via a bilingual questionnaire tailored to this population. The results highlight significant associations between physical activity levels and factors such as age, employment, education, marital status, and perceived control over psychological stressors, with differences observed between men and women. The study concludes that these findings could inform targeted health promotion strategies to improve physical activity among Resettlers, considering their unique challenges and circumstances.

While the study is well-constructed and offers important insights, several areas require refinement to enhance the paper's clarity, coherence, and overall impact.

Specific comments:

1. The introduction effectively outlines the issue of physical inactivity, but the articulation of the specific research gap could be stronger. It would benefit from a more explicit explanation of why the focus on Resettlers is critical and how this study addresses existing gaps in the literature.

2. The transition between the global context, the situation in Germany, and the focus on Resettlers is somewhat abrupt. Smoother transitions and clearer connections between these sections would help guide the reader through the rationale behind the study.

3. The methodology section is thorough, but the decision to use self-reported physical activity assessments needs a stronger justification, especially considering their known limitations. While the study acknowledges the limitation of self-reported data, it would be beneficial to discuss how missing data, particularly in sections like frequent pain (with 12.3% missing data), was handled and the potential impact this could have on the study's findings.

4. The statistical analyses are presented in detail, but some interpretations lack depth, particularly regarding the implications of the findings. The discussion could be enhanced by providing a more nuanced interpretation of how these results contribute to our understanding of physical activity among Resettlers.

5. The R^2 values reported in the multivariate linear regression models vary, with some models showing relatively low R^2 values, especially for women's physical activity. This suggests that the models may not fully capture the variability in the data. It would be beneficial to discuss the potential limitations of these models and consider whether additional variables or alternative modeling approaches could improve the model fit.

6. The manuscript mentions the use of backward selection in the regression models. While this is a valid approach, it's important to briefly justify why backward selection was chosen over other selection methods. A discussion on how this choice impacts the robustness and generalizability of the models would strengthen the analysis.

7. The analysis is disaggregated by sex, which is an important and commendable approach. However, the manuscript should consider addressing whether interaction effects between sex and other variables (e.g., age, employment status) were explored. Including interaction terms in the regression models could provide deeper insights into how these factors jointly influence physical activity.

8. The handling of missing data, particularly in the context of the statistical models, is crucial. The manuscript should clearly state whether any imputation methods were used, or if the analysis was conducted only with complete cases. If the latter, the potential biases introduced by excluding cases with missing data should be discussed.

9. The paper touches on gender differences in physical activity, which is a crucial aspect, but it could delve deeper into the cultural and social factors that might explain these differences.

10. The conclusion of the study summarizes the findings well but could be more impactful by clearly stating the practical implications of the research. It would be beneficial to emphasize how these findings could inform public health policies or interventions aimed at improving physical activity levels among Resettlers and other migrant groups.

11. The study mentions that the questionnaires were bilingual and tailored to the subpopulation under study, which is commendable. However, more detail on how this tailoring was done and its effectiveness in capturing accurate data would strengthen the methodology section.

12. While the study provides a broad overview of the factors influencing physical activity among Resettlers, it could benefit from a more focused analysis on the interplay between these factors and how they differ from the native German population.

13. The manuscript does a good job of highlighting the unique challenges faced by Resettlers in maintaining physical activity. However, incorporating more recent literature on the topic would ensure that the discussion is grounded in the latest research and trends.

VERSION 1 - AUTHOR RESPONSE

Reviewer's Comments to Author:

1. The introduction effectively outlines the issue of physical inactivity, but the articulation of the specific research gap could be stronger. It would benefit from a more explicit explanation of why the focus on Resettlers is critical and how this study addresses existing gaps in the literature.

   Response: We appreciate your critique of the introduction section. Your feedback is valuable in helping us refine our message on migration and physical activity. We have taken your suggestions on board and have tried to reframe the introduction to better highlight the study's importance in addressing the issue of physical inactivity among Resettlers and its contribution to the broader research agenda on Resettler's health. Additions to the introduction section can be found in lines 117-124, 127-130, and 133-138.

2. The transition between the global context, the situation in Germany, and the focus on Resettlers is somewhat abrupt. Smoother transitions and clearer connections between these sections would help guide the reader through the rationale behind the study.

   Response: In response to this comment, we have revised the flow. We have reformulated the text to align with the definition of a migrant, providing a comprehensive view of the impact of their disease burden and its connection to physical activity. Additionally, we have highlighted the disparities in physical activity between autochthonous communities and migrants, suggesting a need to explore the social determinants of health, related to migration. This points to a potential area for future investigation in the Resettler population, considering their disease burden, the lack of literature, and the novelty of social determinants of health.

3. The methodology section is thorough, but the decision to use self-reported physical activity assessments needs a stronger justification, especially considering their known limitations. While the study acknowledges the limitation of self-reported data, it would be beneficial to discuss how missing data, particularly in sections like frequent pain (with 12.3% missing data), was handled and the potential impact this could have on the study's findings.

   Response: In relation to the measurement of physical activity, no justification was given for using subjective measures. This was an oversight; additional information has been included in lines 183-185. Furthermore, details on handling missing cases have been added to the analysis section in line 222. Moreover, a particular reason why we had missing cases for “frequent pain” and the potential impact of missing cases is further discussed in the discussion section “methodological limitation” in lines 432- 437.

4. The statistical analyses are presented in detail, but some interpretations lack depth, particularly regarding the implications of the findings. The discussion could be enhanced by providing a more nuanced interpretation of how these results contribute to our understanding of physical activity among Resettlers. comprehensive

Response: The discussion section, lines 392-406, now includes a section on the research implications.

5. The R2 values reported in the multivariate linear regression models vary, with some models showing relatively low R^2 values, especially for women's physical activity. This suggests that the models may not fully capture the variability in the data. It would be beneficial to discuss the potential limitations of these models and consider whether additional variables or alternative modeling approaches could improve the model fit.

   Response: Thank you for this observation. We have discussed this as a potential limitation in our study, including calling for more robust research targeting Resettlers to investigate the associations with physical activity. Additions have been made in the discussion section "Methodological limitations" lines 423-431.

6. The manuscript mentions the use of backward selection in the regression models. While this is a valid approach, it's important to briefly justify why backward selection was chosen over other selection methods. A discussion on how this choice impacts the robustness and generalizability of the models would strengthen the analysis.

   Response: Forward selection is usually superior in defining good models in case of larger numbers of covariables because it does not use the full set of variables, which could yield model instability. On the other hand, starting with the full model, that is, backward selection, allows us to deal better with collinearity between variables. Ultimately, none of the selection models consider causal relationships or care for confounders. However, as we were interested in models that explain the physical activity pattern best rather than being interested in the impact of a single variable (adjusted for confounders), we think that stepwise backward selection is a valid approach here to get a first idea about the predictors for physical activity among Resettlers. Before using backward selection, we fitted a regression model adjusting for all variables and found no relevant difference between the full and backward selection models.

Additions to the justification for a backward selection have been made to the analysis section in lines 222-227.

7. The analysis is disaggregated by sex, which is an important and commendable approach. However, the manuscript should consider addressing whether interaction effects between sex and other variables (e.g., age, employment status) were explored. Including interaction terms in the regression models could provide deeper insights into how these factors jointly influence physical activity.

   Response: We appreciate this comment as it offers an insightful approach to further our analysis of physical activity among Resettlers. We are, however, keen to emphasize that as a first step, due to the dearth of literature on this topic, our objective aimed to analyze sex differences in the different layers of associations from a biomedical and social determinants standpoint to further give insights into further analyses, e.g., the moderations sex has on key independent significant variables, as you have mentioned. Additionally, due to the limited sample size, we decided to run disaggregated models for women and men instead of including interaction terms in a combined model. As mentioned, we aimed to get a first idea about prognostic factors, but the dataset hardly allows deeper investigation. The sample size might be too small and have limited power to detect interaction effects.

8. The handling of missing data, particularly in the context of the statistical models, is crucial. The manuscript should clearly state whether any imputation methods were used, or if the analysis was conducted only with complete cases. If the latter, the potential biases introduced by excluding cases with missing data should be discussed.

   Response: Lines 432- 437 of the “methodological limitations” section under the discussion section have been added.

9. The paper touches on gender differences in physical activity, which is a crucial aspect, but it could delve deeper into the cultural and social factors that might explain these differences.

Response: We have improved to highlight the sex differences and accredited some of these differences to cultural imprints in the demographic profiles, psycho-social factors, and health behavior associations with physical activity. These can be found on the line…319-326, 344-349, 364, 381-382, and 388.

10. The conclusion of the study summarizes the findings well but could be more impactful by clearly stating the practical implications of the research. It would be beneficial to emphasize how these findings could inform public health policies or interventions aimed at improving physical activity levels among Resettlers and other migrant groups.

    Response: Thank you for this suggestion. We have considered it and added lines 403-406 and 568-572.

11. The study mentions that the questionnaires were bilingual and tailored to the subpopulation under study, which is commendable. However, more detail on how this tailoring was done and its effectiveness in capturing accurate data would strengthen the methodology section.

    Response: We have now clearly mentioned this in lines 204-208

12. While the study provides a broad overview of the factors influencing physical activity among Resettlers, it could benefit from a more focused analysis on the interplay between these factors and how they differ from the native German population.

    Response: We appreciate this comment; this addition would have made an excellent analysis. However, our sample population is entirely Resettlers from the former Soviet Union; as such, we were/are unable to explore such an analysis.

13. The manuscript does a good job of highlighting the unique challenges faced by Resettlers in maintaining physical activity. However, incorporating more recent literature on the topic would ensure that the discussion is grounded in the latest research and trends

    Response: Thank you for this quite valid criticism. However, owing to our focus on building a comparison of our sample with migrants, migrants of European descent, or persons living in

the FSU to highlight the impact of individual biographies and their associations with physical activity, we ended up having a mix of current and old citations. Getting updated literature on this topic was quite a challenge due to the dearth of research. Regardless, we have tried to update the following citations in the discussion section: 31,40, 49,52, 59-62, and 74-82.

VERSION 2 - REVIEW

Reviewer 1

Name Jung, Myungjin

Affiliation University of North Texas, Kinesiology, Health Promotion, and Recreation

Date 03-Nov-2024

COI

The authors have done a good job of responding to the concerns that were raised during the review process. I would be happy to see this published. The additional content that has been added has strengthened the scope and context of this work.
